# A two-week exercise intervention improves cold symptoms and sleep condition in cold-sensitive women

**DOI:** 10.1186/s40101-023-00339-y

**Published:** 2023-09-29

**Authors:** Fumio Yamazaki, Kana Inoue, Nanako Ohmi, Chika Okimoto

**Affiliations:** Department of Nursing, Faculty of Nursing and Human Nutrition, Yamaguchi Prefectural University, 6-2-1 Sakurabatake, Yamaguchi, 753-0021 Japan

**Keywords:** Cold symptom, Aerobic exercise, Sleep, Thermal sensation, Skin temperature

## Abstract

**Background:**

We examined whether an aerobic exercise intervention in young women with cold sensitivity symptoms improves sleep quality and decreases cold complaints. Furthermore, we examined the association with increased foot skin temperature (Tsk) before falling asleep and decrease in sensitivity to cold in the brain.

**Methods:**

We recruited 16 female adult volunteers who had cold sensitivity and were not engaged in daily exercise training, and they were divided into an exercise group (EXE) and a control group (CON). EXE was given a 2-week exercise intervention that consisted mainly of walking and jogging. Before and after the intervention, temperature sensation and body temperature parameters were measured just before bedtime; electroencephalogram measurements were taken during sleep; and subjective sleep surveys, including Oguri-Shirakawa-Azumi (OSA) sleep inventory (middle-aged and aged version) and visual analogue scale (VAS), were conducted immediately after waking up. All experiments were performed in the winter season.

**Results:**

In EXE, overall and foot warmth and comfort increased (*p* < 0.05) after the 2-week exercise intervention. The exercise intervention also decreased (*p* < 0.05) the scores for cold feeling in the fingertips, feet, and toes. In the OSA sleep inventory, factor IV (refreshing) and factor V (sleep length) were increased (*p* < 0.05) by the exercise intervention. Subjective sleep quality evaluated by VAS increased (*p* < 0.05) with exercise intervention. The exercise intervention in EXE shortened middle awake time after sleep onset (*p* < 0.05) and prolonged deep sleep length (*p* < 0.05). The exercise intervention increased (*p* < 0.05) alpha-wave power before sleep. In CON, all variables remained unchanged throughout the 2-week control period.

**Conclusion:**

Short-term aerobic exercise alleviated peripheral extremity cold sensitivity symptoms and improved subjective sleep quality. Our findings suggest that these improvements were not due to increased Tsk at rest before bedtime but to decreased sensitivity to cold in the brain that was expressed as increased alpha activity.

## Background

Thermal comfort feeling exhibits inter-individual differences. A cold constitution with higher sensitivity to cold sensations is called “*hi-e-sho*” in Japanese. This condition is more common in females than in males [1–3]. Previous studies have reported that Japanese females experience severe chilliness even under temperate thermal conditions, especially in the lower extremities [1–5]. Cold in the lower extremities hinders the body’s recovery from sleep, which is associated with poor sleep onset and a decrease in the feeling of sound sleep [6, 7]. Thus, thermal discomfort and insomnia due to cold sensitivity in daily life reduce quality of life.

A previous study reported that when falling asleep, an increase in skin temperature (Tsk) of the peripheral extremities and a decrease in metabolic rate decreased core body temperature [8]. Tsk acts as an input signal to the sleep regulation system, and increased Tsk shortens sleep onset latency [9]. In people with sensitivity to cold, increasing foot Tsk by heating the lower extremities with a far-infrared heater before bedtime improves sleep quality and promotes recovery from fatigue [10]. Recently, Yamazaki et al. [11] reported that daily aerobic exercise over a 4-week period was effective in alleviating peripheral cold symptoms in the extremities and suggested that the involved physiological factors were reduced cold sensitivity in the brain and increased cutaneous vasodilation in the feet. These studies suggest that the promotion of blood circulation through lower extremity heating increases Tsk and has a positive effect on sleep quality by promoting sleep onset; thus, aerobic interventions, such as walking or jogging, that promote blood circulation in lower extremity muscles may be effective in improving cold and sleep.

Previous studies examining the relationship between sleep and chronic exercise in healthy adults suggested a favorable effect of exercise training on sleep [12–16]. Male army soldiers who participated in an 18-week training program had improved sleep, particularly increased slow-wave sleep [15]. On the other hand, in young women with no sleep problems, improving aerobic fitness with a 12-week endurance training program had no effect on sleep [14].

Recently, we reported that electroencephalograms (EEG) of people with sensitivity to cold were characterized by lower alpha-wave power during eye closure at room temperature than noncold sufferers [17]. We also reported that alpha-wave power was significantly negatively correlated with the degree of sensitivity to cold, whereas beta-wave power at rest with eyes closed showed a significant positive correlation [18].

This study aimed to determine the effects of a short-term aerobic exercise intervention on sleep status and cold sensory function in young women with cold complaints. We also assessed whether the aerobic exercise intervention improved subjective and objective sleep quality with a decrease in cold complaints, and whether this was associated with increased foot Tsk and decreased cold sensation prior to sleep onset. Furthermore, we examined whether the aerobic exercise intervention increased alpha-wave power and decreased beta-wave power with a decrease in cold discomfort, which would be associated with improved sleep.

## Methods

### Subjects

The subjects were 16 healthy females aged 20–21 who had sensitivity to cold. The subjects had not regularly engaged in exercise or sports activities in their daily lives for at least the past year. The presence or absence of exercise habits was confirmed by interviewing the subjects. The inclusion criteria were answering five or more of a 10-item questionnaire about body coldness and being aware of sensitivity to cold [19]. The reliability and validity of this questionnaire in screening women with cold sensitivity were confirmed in a previous study [20]. According to its inclusion criteria, 35.7% of young women were judged to have sensitivity to cold, which was similar to previously reported rates [20]. The subjects were randomly divided into an exercise group (EXE) (*n* = 8) who received exercise intervention and a control group (CON) (*n* = 8) who received no intervention in the order of their application. Height (EXE, 161.1 ± 4.5; CON, 156.3 ± 4.0 cm), weight (52.8 ± 6.2; 49.6 ± 3.8 kg, respectively), and body mass index (20.3 ± 1.5; 20.3 ± 1.0, respectively) were similar between the groups. There were no differences between the groups in sleep status in the month before the intervention using the Japanese version of the Pittsburgh Sleep Quality Index (PSQI-J) (EXE, 5.6 ± 1.5 and CON, 4.9 ± 2.1). None of the subjects had been diagnosed with insomnia; however, three in EXE and three in CON had PSQI scores of 6 or higher, which is considered a possible sleep problem. The daily sleep habits were also confirmed by interviewing the subjects.

This study was implemented after the approval by the Bioethics Committee of Yamaguchi Prefectural University (Approval No. 2021–26). Subjects were informed in advance of the purpose, methods, and risks of the experiment, and written consent was obtained. The experimental results are presented as numbers and have been managed under confidentiality.

### Exercise intervention

EXE received a 2-week exercise intervention. The EXE group exercised at least 4 days per week during the intervention period, walking at least 5000 steps per day more than their average number of steps before the intervention. The subjects in EXE were also instructed to perform moderate exercise consisting of at least 15 min of fast walking or jogging to warm up the body in the walking-based aerobic exercise program. The time period and place for the exercise were decided by the subjects. Except for the exercise, the subjects were asked to maintain the same lifestyle during the exercise intervention as before the intervention. In CON, the subjects maintained the same physical activity level as before the intervention.

### Experimental conditions and procedures

EEG, subjective sleep state, thermal sensation, thermal comfort, Tsk, core body temperature, and room temperature were measured on the 2 days immediately before and 2 days immediately after the intervention period at the subject’s home. The variables were measured twice (2 days before and after the intervention) and averaged over the 2 days. Thermal sensation, thermal comfort, and temperature variables were measured daily during the experimental period. An air conditioner was used to adjust the room temperature in the bedroom to 18 °C at the time of measurement. Subjects were asked to go to bed between 23:00 and 1:00, to take a bath at least 1 h before bedtime and to soak for no more than 10 min, to eat at least 3 h before bedtime, not to drink alcohol, and to use the same clothing and bedding on the days examined before and after the intervention period. All experiments were conducted in winter (December to February) when cold complaints are likely to occur.

### Measurements

#### Physical activity

All subjects wore a physical activity meter with a built-in three-axis accelerometer (MTN-221, ACOS Inc., Nagano, Japan) except for bathing time, and their physical activity was continuously recorded from 3 days before to 14 days during the intervention period and 2 days after the intervention period (19 days in total). The number of steps and amount of physical activity were analyzed using a dedicated program. On the days when they exercised, the content of the exercise was recorded on a recording form.

#### Tsk, core body temperature, and room temperature

An infrared thermometer (NIR-10, CUSTOM Inc., Tokyo, Japan) was used for temperature measurement. By switching the measurement mode, this device can instantaneously estimate core body temperature (setting mode: oral temperature) from forehead surface temperature, Tsk, and room temperature with a minimum display temperature of 0.1 °C. The accuracy of measurements was ± 0.3 °C or less. The thermometer was left in a room at 18 °C for at least 10 min for room temperature calibration. Throughout the experimental period, Tsk of the left and right toes and the dorsum of the feet, room temperature, and core body temperature were measured before entering the bed. Foot Tsk was obtained from the average of the left and right Tsk of the toes and dorsum of the feet.

#### Thermal sensation and thermal comfort

Thermal sensation and thermal comfort were measured immediately before bedding throughout the experimental period using a visual analogue scale (VAS) [19]. Subjects were asked to report thermal sensation for the feet and whole body by marking on a 15-cm line rating scale, which was labeled “cold” 2.5 cm from the left end and “warm” 2.5 cm from the right end. We instructed the subjects to mark on the scale how strongly they experienced the sensation of coldness or warmth. In addition, the subjects were allowed to mark the level of thermal sensation beyond the cold or warm point, if necessary. The scales were labeled “discomfort” and “comfort” instead of “cold” and “warm”. Then, the length from the point of discomfort to the marked point was measured as the rating score of thermal sensation or thermal comfort, and presented as a positive value.

A questionnaire survey concerning thermal sensation in 11 regions of the body (arm, abdomen, hand, fingertip, foot, toe tip, neck, shoulder, back, lower back, and buttocks) was performed immediately before sleep, and before and after the intervention. Thermal sensation was scored using the following scale: “3. feel strong coldness”, “2. feel coldness”, “1. feel coldness a little”, “0. not at all”, “ − 1. feel hotness a little”, “ − 2. feel hotness”, and “ − 3. feel strong hotness”.

#### Sleep questionnaires

Subjective sleep quality was evaluated using the Oguri-Shirakawa-Azumi (OSA) sleep inventory (middle-aged and aged version) immediately after waking up [21]. This is a self-reported questionnaire composed of 16 items each with a 4-point scale. The items are consolidated into five subscales: factor I “sleepiness on rising”, factor II “initiation and maintenance of sleep”, factor III “frequent dreaming”, factor IV “refreshing”, and factor V “sleep length”. The Zi value was calculated with higher values indicating better sleep quality. Subjects were also asked to report subjective sleep quality for the exercise intervention using VAS. A 15-cm line rating scale, which was labeled “did not sleep at all” 2.5 cm from the left end and “slept very well” 2.5 cm from the right end was used. In both groups, sleep surveys using the OSA sleep questionnaire and VAS were performed twice before and after the intervention.

#### Sleep EEG

In order to objectively evaluate the state of nocturnal sleep before and after intervention, EEG was recorded from just before lights out to the time of waking using a portable recording system (Sensor ZA-X, Proassist Ltd., Osaka, Japan) [22]. The EEG sensor consists of a telemeter and receiver of a bioamplifier (sampling rate 128 Hz, frequency response 0.5 to 40 Hz). One channel is for monitoring EEG with electrodes on the right forehead and left neck (Fp2-M1), and the second channel is for monitoring electro-oculogram (EOG) and electromyogram with electrodes on the orbit and submental muscles. In all conditions of the experiment, sleep EEG was performed four times, twice on 2 days before and after the intervention.

EEG data was automatically analyzed using a sleep analysis research program (SleepSign-Lite, KISSEI COMTEC Ltd., Nagano, Japan) and the waveforms were evaluated with EOG by visual inspection, which has been reported to be highly reliable [22, 23]. Sleep stages were analyzed by dividing them into waking, rapid eye movement (REM), light sleep (stages N1 and N2), and deep sleep (stage N3) according to the AASM manual for scoring rules Ver. 2.4 [24]. Each sleep stage was carefully checked and corrected by the authors after automatic analysis. The REM sleep phase was determined from eye movements and beta waves. Stages N1 and N2 were grouped together as the light sleep stage: stage N1 was determined as the point at which alpha waves were less than 50% occupied and theta waves became prominent and stage N2 was determined using spindle waves or K-complex as indicators. Stage N3 was determined as a high amplitude delta wave occupancy of 20% or more.

In addition to the analysis of sleep stages, power spectral values of alpha waves (8–12 Hz) and beta waves (16–25 Hz) were analyzed using the sleep analysis research program. The power spectral values were averaged during wakefulness for 2–3 min immediately before sleep and throughout sleeping time. The values were presented as percentages of spectral power of frequency band of 1–25 Hz. In addition, intervention-induced changes in power spectral values were expressed as a percentage change from the pre-intervention baseline.

### Statistical analysis

Means ± SD of all measurement items were calculated. A repeated two-way analysis of variance was performed to test the difference between the experimental conditions in each measurement item, and Bonferroni multiple comparison test was performed as a post hoc test when the interaction was significant. An unpaired *t* test was used to test the difference in pre-intervention means. A level of less than 5% (*p* < 0.05) was considered significant.

## Results

All subjects completed the experiments. The subjects’ diaries on the timing of sleep, bathing, and meals during the experiment period showed that they were almost the same in EXE and CON.

### Physical activity

The number of steps and amount of physical activity per day before the intervention was similar between EXE (3965 ± 1178 steps/day, 291 ± 88 kcal/day) and CON (3581 ± 1617 steps/day, 253 ± 90 kcal/day). The number of steps and the amount of physical activity, including fast walking and jogging, during the intervention in EXE significantly increased (interaction effect, *p* < 0.05; post hoc, *p* < 0.05) from the pre-intervention level by 3652 ± 2080 steps/day and 155 ± 71 kcal/day, respectively. The exercise intervention-induced changes did not differ between the first week (number of steps, + 4105 ± 2713 steps/day; amount of physical activity, + 170 ± 96 kcal/day) and the second week (+ 3133 ± 1574 steps/day; + 137 ± 53 kcal/day) of the exercise intervention. No significant changes (+ 269 ± 2169 steps/day, + 28 ± 105 kcal/day) were observed in both the number of steps and the amount of physical activity during the intervention period from the pre-intervention levels in CON.

### Thermal sensation and thermal comfort

**Fig. 1:**
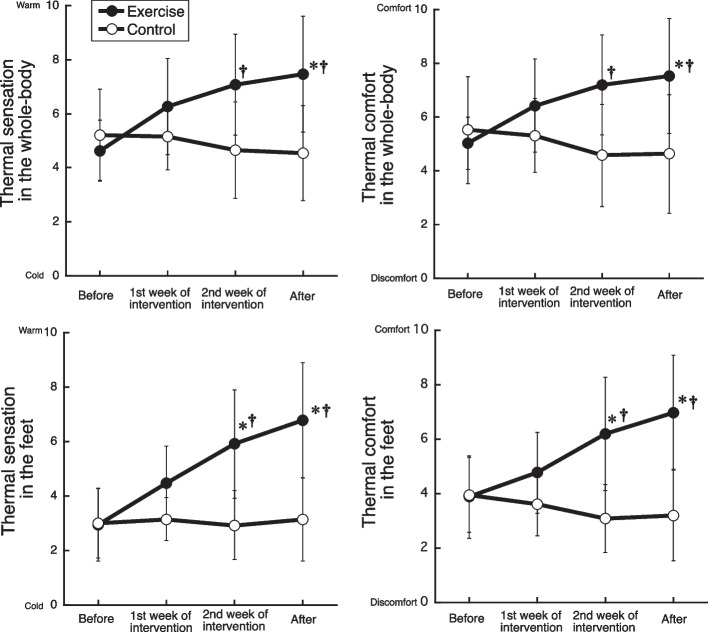
Changes in thermal sensation and thermal comfort in the whole-body and feet with 2-week exercise or time-controlled interventions. *p < 0.05 vs. before, †*p* < 0.05 vs. control

Figure 1 shows changes in thermal sensation and thermal comfort associated with exercise intervention. None of the parameters showed significant differences in pre-intervention values between groups. The whole-body and feet warmth sensations and comfort feelings gradually increased during the intervention in EXE, whereas neither of these variables changed significantly throughout the intervention period in CON. Both the whole-body and feet warmth sensations and comfort feelings were higher (interaction effect, *p* < 0.05; post hoc, *p* < 0.05) in EXE than CON at the second week of intervention and the post-intervention periods.

**Fig. 2:**
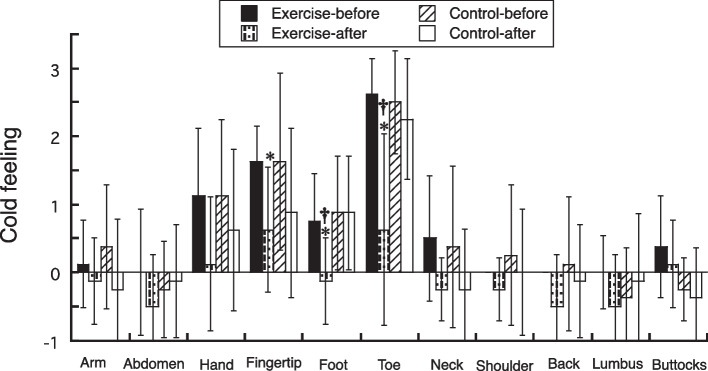
Changes in cold feeling in each region with 2-week exercise or time-controlled interventions. *p < 0.05 vs. before, †*p* < 0.05 vs. control group

Figure 2 shows the cold sensations by body part before and after the interventions by group. The sensations of coldness in the fingertips, feet, and toes decreased (interaction effect, *p* < 0.05; post hoc, *p* < 0.05) with the exercise intervention, and EXE had lower (post hoc, *p* < 0.05) coldness in the feet and toes after the intervention than CON. On the other hand, the exercise intervention did not alter the sensations of coldness in the arms, abdomen, hands, neck, shoulders, back, hips, and buttocks. In CON, the thermal sensations at all sites remained unchanged throughout the time-controlled intervention period.

### Foot Tsk and estimated core body temperature

Before the intervention period, no significant differences were found between groups in foot Tsk (EXE, 24.5 ± 3.0 °C; CON, 25.4 ± 3.2 °C) and estimated core body temperature (EXE, 36.6 ± 0.2 °C; CON, 36.3 ± 0.3 °C). No significant changes in foot Tsk and estimated core body temperature were observed throughout the intervention period in either group. Room temperature at time of body temperature measurement was maintained at a constant level throughout the experimental period. No significant differences were found between groups in room temperature (EXE, 18.9 ± 1.5 °C; CON, 18.3 ± 3.0 °C).

### Sleep questionnaires and sleep EEG

The evaluation results of sleep conditions using the OSA Sleep Inventory are shown in Table 1. There were no significant differences between groups in the pre-intervention values for any of the five factors. In EXE, the intervention increased (interaction effect, *p* < 0.05; post hoc, *p* < 0.05) factor IV (refreshing) and factor V (sleep length); however, no significant changes were observed in either factor in CON.

**Table 1:** Evaluation of subjective sleep quality using the Oguri–Shirakawa–Azumi sleep inventory, middle-aged and aged version, before and after interventions in the exercise and control groups

	Exercise		Control	
	Before	After	Before	After
Factor I (sleepiness on rising)	51.5 ± 6.6	55.8 ± 5.0	45.7 ± 5.7	44.3 ± 6.1
Factor II (initiation and maintenance of sleep)	42.9 ± 13.4	51.7 ± 12.1	41.0 ± 8.7	44.1 ± 11.2
Factor III (frequent dreaming)	49.4 ± 14.4	54.7 ± 5.4	43.4 ± 12.4	43.9 ± 14.5
Factor IV (refreshing)	43.6 ± 14.4	56.2 ± 7.8*	46.9 ± 9.5	46.1 ± 8.5
Factor V (sleep length)	44.0 ± 6.6	58.8 ± 9.6*	51.7 ± 8.6	47.0 ± 9.1

^*^p < 0.05 vs. before

**Fig. 3:**
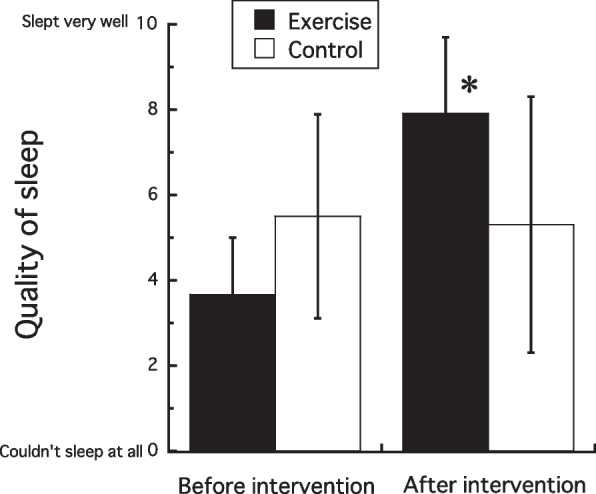
Changes in subjective evaluations of quality of sleep with 2-week exercise or time-controlled intervention. *p < 0.05 vs. before intervention

Figure 3 shows sleep quality evaluated using VAS before and after the interventions. There was no significant difference between groups in pre-intervention sleep quality. The intervention increased (interaction effect, *p* < 0.01; post hoc, *p* < 0.0001) sleep quality in EXE (+ 4.3 ± 2.9), whereas no significant difference was observed in CON before and after the intervention period (− 0.2 ± 1.3). Post-intervention sleep quality tended to be higher in EXE than in CON (post hoc, *p* = 0.05).

There was no difference between the groups in the pre-intervention values for any of the sleep variables by analyzing EEG (Table 2). Analysis of sleep EEG revealed that the exercise intervention shortened (interaction effect, *p* < 0.05; post hoc, *p* < 0.05) wake after sleep onset time and prolonged (interaction effect, *p* < 0.05; post hoc, *p* < 0.05) deep sleep time. No other sleep variables were significantly different before and after the intervention and between groups. In addition, bedtime was not different before (EXE, 0:15 ± 0:31; CON, 0:03 ± 0:49) and after (EXE, 0:27 ± 0:35; CON, 23:45 ± 0:49) the intervention in both groups.

**Table 2:** Analysis of sleep variables using EEG and EOG recording systems before and after interventions in the exercise and control groups

	Exercise		Control	
	Before	After	Before	After
Time in bed, min	402.4 ± 50.6	431.6 ± 83.5	399.1 ± 77.1	424.8 ± 56.9
Total sleep time, min	384.5 ± 53.0	421.5 ± 82.3	388.9 ± 78.4	413.3 ± 56.5
Wake after sleep onset, min	10.3 ± 9.7	2.4 ± 3.3 *	5.9 ± 5.7	3.6 ± 3.6
Sleep latency, min	6.1 ± 6.4	6.9 ± 5.6	5.2 ± 1.8	6.0 ± 3.8
Wake total time, min	12.6 ± 10.7	10.1 ± 5.7	15.8 ± 12.6	14.9 ± 10.3
REM total time, min	66.8 ± 14.1	78.4 ± 8.9	64.0 ± 17.0	80.5 ± 21.2
NREM total time, min	317.2 ± 41.8	343.0 ± 78.7	324.9 ± 69.7	332.9 ± 47.9
Light total time (stages N1 + N2), min	263.2 ± 33.1	258.8 ± 62.9	273.0 ± 64.9	276.6 ± 42.4
Deeply total time (stage N3), min	54.1 ± 16.9	84.3 ± 22.2 *#	51.8 ± 29.2	56.3 ± 36.5
Sleep efficiency, %	95.4 ± 3.9	97.6 ± 1.3	96.4 ± 2.9	97.3 ± 1.1

*EEG* electroencephalography, *EOG* electro-oculography, *REM* rapid eye movement, *NREM* non-REM

^*^p < 0.05 vs. before

^#^*p* < 0.05 vs. control

The exercise intervention increased (interaction effect, *p* < 0.05; post hoc, *p* < 0.05) alpha-wave power during pre-sleep arousal by 71.1 ± 48.4% from pre-intervention values but did not change the average value (− 2.0 ± 25.6%) during sleep (Table 3). There was no significant difference in alpha-wave power before and after the time-controlled intervention period in the CON group. Neither beta-wave power on awakening before falling asleep nor the average values during sleep were altered by the exercise intervention.

**Table 3:** Changes in spectral power of alpha- and beta-bands before and after interventions in the exercise and control groups

	Exercise		Control	
	Before	After	Before	After
Alpha-wave power before sleep, %	7.9 ± 6.3	12.5 ± 10.3 *	7.3 ± 4.5	7.0 ± 3.9
Beta-wave power before sleep, %	2.5 ± 2.0	3.8 ± 3.0	1.6 ± 0.9	1.9 ± 1.2
Alpha-wave power during sleep, %	4.4 ± 0.9	4.3 ± 1.4	4.3 ± 2.5	4.4 ± 2.2
Beta-wave power during sleep, %	1.5 ± 1.0	1.4 ± 1.0	1.2 ± 0.7	1.2 ± 0.9

^*^p < 0.05 vs. before

## Discussion

This is the first study to investigate the effects of an aerobic exercise intervention on sleep state and thermosensory function in people with sensitivity to cold. Short-term aerobic exercise, such as walking and jogging, alleviated coldness of the extremities in the normal temperature environment, which was considered to be due to the decrease in cold sensation. The aerobic exercise intervention improved subjective sleep quality, which was thought to be associated with shorter wakefulness and longer deep sleep.

### Thermal sensation and comfort feeling

Previous studies reported reduced effects of coldness in extremities in young women by 4-week aerobic exercise intervention [11] and by 3-month mixed aerobic and resistance training [25]. In the present study, thermal sensation and comfort of the whole body and feet increased gradually during the exercise intervention, and both values significantly increased 2 weeks after the intervention compared to the pre-intervention values. As there was no significant increase in comfort feelings during the first week of the exercise intervention, our findings suggest that 1 week is too short a time period to reduce thermal discomfort in people with sensitivity to cold under the experimental condition.

In prior studies, the relationship between sensitivity to cold and exercise habits was examined by comparing coldness between those with and without exercise habits. An internet survey of 6729 patients reported that those who were less physically active were more likely to be aware of cold [26]. In a study of 212 female college students, Tsuyushige et al. [27] reported that cold symptoms were milder in those who performed physical exercise on a daily basis than in those with no exercise habits. These prior studies were cross-sectional studies that compared those with and without exercise habits. The present study was a randomized controlled trial intervention study that found that a 2-week short-term exercise intervention was effective in reducing coldness in women with sensitivity to cold.

When changes in coldness were examined by body part (Fig. 2), coldness in the fingertips, feet, and toes decreased with the exercise intervention, and the EXE group had significantly lower coldness in their feet and toes after the intervention than the CON group. We found that aerobic exercise was effective in relieving cold symptoms in the peripheral extremities, but not in the trunk. These findings support a previous study that reported that walking exercise decreased cold sensitivity in the brain and increased the cutaneous vasodilator response in the feet [11]. As the peripheral extremities are more susceptible to cold prior to intervention, the effect of exercise on alleviating cold symptoms may be more readily apparent than the trunk.

We hypothesized that an increased core temperature and an enhanced blood flow in the skin during exercise would alleviate cold symptoms. In a previous study of cold-sensitive women, we reported that (1) the discomfort feeling during hand cooling was decreased by the increase in core body temperature associated with 15 min of leg exercise, and (2) the pattern of changes in cold sensation after exercise varied due to differences in Tsk increase in different body regions [28]. Thus, a single bout of exercise produced a transient decrease in thermal discomfort, both systemically due to an increase in core body temperature and regionally due to different increases in Tsk by part; however, once body temperature was restored to pre-exercise levels, the warm and cold sensations also returned to normal. The 2-week exercise intervention did not change the estimated core body temperature and foot Tsk during rest at normal resting temperatures. These findings were consistent with a previous study in which body temperature was measured by thermistor thermometer before and after a 4-week aerobic exercise intervention [11]. Thus, there were significant changes in thermal sensation and discomfort that were considered to be due to decreased sensitivity to cold and discomfort under normal temperature conditions.

### Changes in sleep status

In the OSA Sleep Inventory used in this study, factor IV (refreshing) and factor V (sleep length) increased with exercise intervention. The exercise intervention also increased sleep quality in subjective sleep assessment using VAS. The effectiveness of physical activity as one of the preventive measures for insomnia and sleep disorders has been reported in previous studies [29, 30]. Inoue et al. [31] conducted a 2-year longitudinal study of 3679 community-dwelling elderly people to examine the association between exercise habits and sleep, and found that those who exercised at least 5 days per week were less likely to experience mid-wake, one of the symptoms of insomnia, than those who did not exercise, indicating that habitual exercise may help control insomnia symptoms. Kubitz et al. [32] reported in a meta-analysis that both transient and habitual exercise increased deep sleep and total sleep time in healthy subjects with less mid-waking incidents. The present study found that the exercise intervention in EXE resulted in shorter mid-awake times and longer deep sleep times without changing total sleep time (Table 2). Thus, participants with cold sensitivity in EXE were able to continue to sleep deeply at night to recover from the mild fatigue caused by physical activity, which led to a sense of recovery from fatigue and longer subjective sleep duration, which in turn led to higher subjective sleep quality.

Since there was no difference in pre-bedtime foot Tsk before and after the intervention in either group, the improvement in sleep quality due to the exercise intervention was not related to the increase in foot temperature. On the other hand, we found a decreased sensitivity to cold in the peripheral extremities before falling asleep without a change in body temperature. Thus, the decrease in thermal discomfort associated with decreased cold sensitivity may have contributed to the improvement in sleep quality due to the exercise intervention.

Alpha activity in EEG is increased during relaxing and eye-closed resting, and is decreased as a result of aging and pathological states [33–35]. In healthy young adults, the degree of cold sensitivity is significantly negatively correlated with values of alpha-wave power during eye-closed resting under normal temperature conditions, whereas it is significantly positively correlated with values of beta-wave power [18]. In subjects with sensitivity to cold, a previous study reported that the spectral power of alpha-waves (ranging from 8 to 10 Hz) was lower than normal control subjects during normothermia and whole-body cooling in the daytime [36]. In that study, since whole-body cooling and rewarming did not acutely change alpha-wave power in both subject groups, it may take a certain period of time for suppression of thermal discomfort associated with decreased cold sensation in the brain to occur, characterized by increased alpha activity during eye-closed resting. The present study showed for the first time that the alleviation of pre-sleep coldness by aerobic exercise practice for 2 weeks in young women with sensitivity to cold occurred with an increase in alpha-wave power, but not with a suppression of beta-wave power. Interventions using yoga exercise [37] and body-mind training [38] also increase physical and mental relaxation, as indicated by decreased serum cortisol and state anxiety and increased alpha waves. These findings suggest that there is a relationship between lower alpha-wave power before sleep onset and cold discomfort. Thus, the thermally induced relaxing effects of light exercise in the daytime may decrease chills and insomnia in the nighttime.

Some considerations are required to interpret the results in this study. First, in the present study, we used an infrared thermometer for temperature measurement. In previous studies, an infrared forehead skin thermometer for core temperature measurements did not provide reliable values of body temperature during periods of increasing body temperature [39] and after cold provocation [40]. Thus, previous studies suggest that thermometry using an infrared thermometer should be used under thermally steady state [39, 41], such as resting state in a constant room temperature; thus, we used these conditions in the present study. One-week endurance training for 2 h per day in a hot environment reduces core body temperature by 0.1–0.5 °C [42], whereas a previous study reported that 4-week aerobic exercise training at light intensity in a normal temperature environment did not alter core body temperature during rest before sleep [11]. These findings suggest that the alleviation in cold sense due to aerobic exercise intervention in the cold-sensitive subjects observed in the present study was not due to an increase in resting body temperature, but rather to a decrease in cold sensitivity at normothermia.

Second, the lifestyle behaviors of subjects were not as strictly controlled as a laboratory sleep experiment. In the present study, we dictated subjects to a bedtime range, timing and method of bathing, and eating as well as monitoring their physical activities. Since the experimental conditions, excluding physical activity, in EXE were similar to the subjects in CON, the response differences between groups in thermal sensation and sleep before and after the intervention period were considered to be due to the effect of exercise itself.

Finally, it was not possible to quantify how much discomfort associated with cold feet before falling asleep was involved in the improvement of sleep quality by aerobic exercise. Thus, it will be necessary to compare the effects of aerobic exercise on sleep and cold sensation before falling asleep, and alpha-wave power in people who do not have sensitivity to cold. Further experiments are needed to investigate whether there is a cutoff value for the percentage of alpha-wave power between cold-sensitive and normal young women.

In conclusion, in cold-sensitive young women, a 2-week aerobic exercise intervention centered on walking and jogging alleviated coldness in the peripheral extremities and improved subjective sleep quality with shorter mid-awake time and longer deep sleep time. These improvements were not due to increased Tsk before sleeping but to decreased discomfort to cold, which was presented as increased alpha-wave power.

## Data Availability

The datasets generated during the current study are not publicly available but are available from the corresponding author on reasonable request.

## Abbreviations

Tsk: Skin temperature
EEG: Electroencephalogram
EOG: Electro-oculogram
EXE: Exercise group
CON: Control group
OSA: Oguri-Shirakawa-Azumi
VAS: Visual analogue scale
PSQI-J: Japanese version of the Pittsburgh Sleep Quality Index
REM: Rapid eye movement
NREM: Non-rapid eye movement
